# Noise Propagation and Signaling Sensitivity in Biological Networks: A Role for Positive Feedback

**DOI:** 10.1371/journal.pcbi.0040008

**Published:** 2008-01-04

**Authors:** Gil Hornung, Naama Barkai

**Affiliations:** 1 Department of Molecular Cell Biology, Weizmann Institute of Science, Rehovot, Israel; 2 Department of Molecular Genetics, Weizmann Institute of Science, Rehovot, Israel; 3 Department of Physics of Complex Systems, Weizmann Institute of Science, Rehovot, Israel; Lawrence Berkeley National Laboratory, United States of America

## Abstract

Interactions between genes and proteins are crucial for efficient processing of internal or external signals, but this connectivity also amplifies stochastic fluctuations by propagating noise between components. Linear (unbranched) cascades were shown to exhibit an interplay between the sensitivity to changes in input signals and the ability to buffer noise. We searched for biological circuits that can maintain signaling sensitivity while minimizing noise propagation, focusing on cases where the noise is characterized by rapid fluctuations. Negative feedback can buffer this type of noise, but this buffering comes at the expense of an even greater reduction in signaling sensitivity. By systematically analyzing three-component circuits, we identify positive feedback as a central motif allowing for the buffering of propagated noise while maintaining sensitivity to long-term changes in input signals. We show analytically that noise reduction in the presence of positive feedback results from improved averaging of rapid fluctuations over time, and discuss in detail a particular implementation in the control of nutrient homeostasis in yeast. As the design of biological networks optimizes for multiple constraints, positive feedback can be used to improve sensitivity without a compromise in the ability to buffer propagated noise.

## Author Summary

Biological circuits need to be sensitive to changes in environmental signals but at the same time buffer rapid fluctuations (noise) that might be imposed on this input. In this paper, we analyze the interplay between sensitivity to signals and the ability to buffer noise. Previous studies reported that negative feedback attenuates noise. We show, however, that this ability comes at the expense of an even more dramatic reduction in sensitivity. In fact, when comparing systems of the same sensitivity, a system with negative feedback is more amenable to noise than a system without such feedback. We searched for small biological circuits that can buffer noise while maintaining high sensitivity, and found that positive feedback exhibits this property. This ability of positive feedback to buffer noise reflects its slowed-down dynamics. We discuss general requirements for the function of positive feedback as a noise-filtering device and describe a particular implementation that appears to function in yeast nutrient homeostasis. Our study emphasizes the need to consider multiple constraints when analyzing the design logic of biological networks.

## Introduction

Cells sense and process information using biochemical networks of interacting genes and proteins. Typically, a signal is sensed at a specific point of the network (input) and is propagated to modulate the activity or abundance of other network components (output). Reliable information processing requires high sensitivity to changes in the input signal but low sensitivity to random fluctuations in the transmitted signal. Since the detection of signal is inherently stochastic [1], and the microenvirnment of the cell is also fluctuating randomly, understanding the principles of noise propagation in biochemical and genetic networks is of interest [2–5].

Linear (unbranched) cascades present the simplest instance of biochemical networks. Recent studies have shown that such cascades display an interplay between sensitivity to changes in input signal and the ability to buffer stochastic fluctuations [6–9]. Indeed, an increase in the sensitivity toward input signals results also in elevated sensitivity to noise in the input. A key question is whether network connectivity, e.g., the presence of positive or negative feedbacks, can modulate this interplay, reducing propagated noise while maintaining high sensitivity. Previous studies argued that negative feedbacks can buffer noise relative to linear cascades [10–12]. These studies, however, did not consider the associated changes in signaling sensitivity.

In general, the fine line that separates “noise” from “signal” is established functionally. Nevertheless, in many systems such as the sensing of temperature, nutrient levels, ligand concentration, etc., the signal is interpreted as a long-term change in the input, whereas noise is characterized by rapid stochastic fluctuations. In this study, we focus on this particular class of systems.

We explore for gene circuits that can buffer propagated noise while maintaining signaling sensitivity. We consider a large set of networks that are differentially designed but are equally sensitive to long-term changes in the input, and compare their ability to buffer propagated noise. Systematic analysis of all three-gene circuits revealed that negative feedback amplifies propagated noise. In contrast, positive feedback appears to be a necessary element for buffering such noise. Analytical analysis demonstrated that positive feedback contributes to noise buffering by slowing down the dynamics, thus providing a longer averaging time. A detailed analysis of a recurrent network design, found in systems controlling nutrient homeostasis, suggests that it functions as a noise-reduction device based on the principles identified in our analysis.

## Results

### Negative Feedback Amplifies Propagated Noise When Sensitivity Is Controlled for

**Figure 1:**
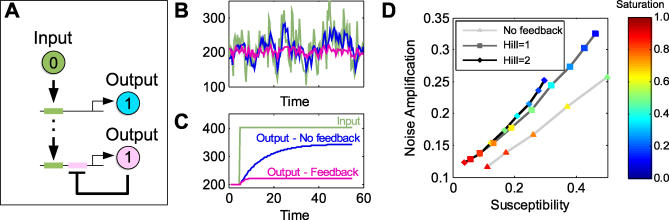
Comparison of a System with Negative Feedback and without Negative Feedback (A) A system with negative feedback (pink) and without negative feedback (blue) were compared in terms of (B) the output noise in response to fluctuations in the input (green), and (C) the deterministic response of the two systems to a 2-fold change in the input. (D) Noise amplification versus susceptibility for negative feedback with different Hill coefficients. The color-code is the level of saturation of the input promoter. Sensitivity was calculated by solving the steady-state equations after a 1% change in the input levels, and noise amplification was the result of stochastic simulations (Methods).

To begin analyzing the effect of network architecture on the interplay between sensitivity and noise buffering, we considered a two-component cascade with a negative feedback loop. This cascade is composed of an input node, *n*_0_, which activates an output node, *n*_1_. The output node feeds back to repress its own expression (Figure 1A). Formally, this system is described by

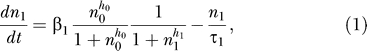
where *β*_1_ denotes the maximal rate of *n_1_* production, 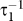 denotes the rate of *n*_1_ degradation, and *h*_0*,*_ *h*_1_ are Hill coefficients. Note that *n*_0_ and *n*_1_ are normalized by their respective dissociation constants from the gene promoter.

We consider an input signal *n*_0_(*t*) = 〈*n*_0_〉 + *σ*_0_(*t*) which fluctuates around some mean level 〈*n*_0_〉. The fluctuating component *σ*_0_(*t*) has a zero mean and some autocorrelation time *τ*_0._ Figure 1B depicts the temporal fluctuations of *n_1_* for a system with a strong negative feedback (Hill coefficient *h*_1_ = 4). The analogous dynamics for a system that lacks such feedback (*h*_1_ = 0) is also shown. Consistent with previous studies [10–12], output noise is lower in the presence of negative feedback. Nevertheless, negative feedback also significantly lowers the sensitivity of the system to a two-fold change in the level of the mean input (Figure 1C).

To rigorously quantify the interplay between the sensitivity of the input–output relation and the buffering of propagated noise, we define two measures for the sensitivity and noise-amplification of the system*.* The steady-state sensitivity is captured by the *susceptibility*, *s*, [3,13,14] (also termed *gain* [9]) defined as the relative change in output following a change in the input:

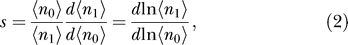
with all quantities measured at steady state. The measure for noise amplification 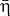 is defined as the ratio between the output and input noise:

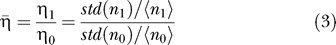



As before, all quantities are measured at steady state. Both *s* and 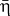 depend on the different parameters of the system, including the Hill coefficients and mean input levels.

Figure 1D depicts the noise amplification versus susceptibility for different levels of mean input. The case of no feedback (*h*_1_ = 0) is compared to that of increasing feedback cooperativity (*h*_1_ = 1 and 2). Again, a clear interplay between susceptibility and noise buffering is observed, with systems that are more sensitive to changes in the input level being also more vulnerable to noise. Notably, this interplay seems to be more severe in the presence of negative feedback. Thus, for a given level of susceptibility, propagated noise is amplified to a greater extent in the presence of a negative feedback. This result is consistent with the theoretical arguments for a two-node model [3]: with negligible intrinsic noise, and when controlling for sensitivity, negative feedback enhances, rather then represses, propagated noise.

### Formulation of a Systematic Screen of All Three-Nodes Networks

**Figure 2:**
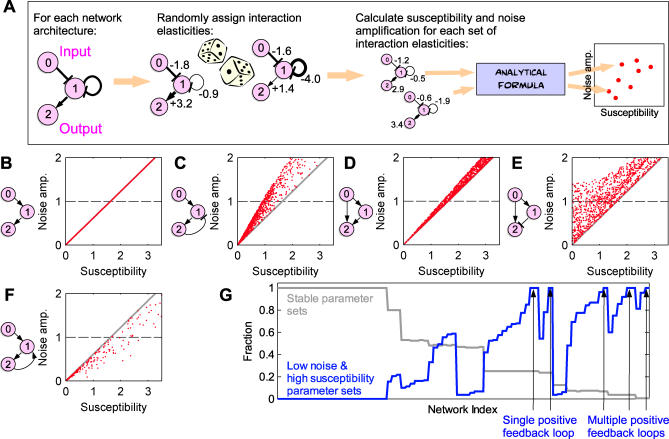
Screen for Networks with High Susceptibility and Low Noise Amplification (A) Each network with an input node 0, an output node 2, and an intermediate node 1 was assigned random sets of interaction elasticities. The susceptibility and noise amplification were calculated from each elasticity set. Results are shown for (B) linear cascade (1,000 points), (C) two-node negative feedback (1,000 points), (D) coherent FFL (1,000 points), (E) incoherent FFL (1,000 points), and (F) two-node positive feedback (230 points with a stable steady state). (G) All networks were sorted into groups according to the fraction of parameter sets that exhibited stability (gray line). Within each group, the networks were sorted according to the fraction of low noise and high susceptibility parameter sets (relative to a linear cascade) out of the stable sets (blue line). As the number of positive feedback loops increases, stability is decreased.

Our analysis implies that negative feedback cannot be used to buffer against rapidly varying propagated noise in systems which require a sensitive response to long-term changes in their input. To identify network architectures that can buffer noise while maintaining sensitivity, we characterized systematically the relation between susceptibility and noise-buffering of all three-node circuits (Figure 2A). A three-node circuit is composed of an input node (*n*_0_), an output node (*n*_2_), and an intermediate node (*n*_1_), connected via activating or repressing interactions (arrows). We allowed for all incoming or outgoing arrows, with the exception of the input *n*_0_ which could affect both *n*_1_ and *n*_2_ (outgoing arrows), but was not subject to feedback regulation (no incoming arrows). Each arrow was assigned a positive sign (activation) or a negative sign (repression), thus leading to a total of 324 networks (Text S1, Section IV). To ensure controlled comparison between networks, we assume that degradation is not regulated and that all proteins degrade at the same rate (e.g., by dilution).

Each specific circuit supports a range of dynamic behaviors, depending on the precise value of the interaction parameters. Following the formalism presented by Paulsson [3,13], we used the Fluctuation Dissipation Theorem (FDT [15]) to derive an analytical formula for susceptibility and noise-amplification (Methods). Briefly, the system of equations that describe the dynamics of the three-node network was

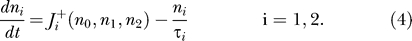



The degradation terms were assumed to be first-order, and we considered *τ_i_* = 1 to maintain a mathematically controlled comparison. This system of equations was linearized around the steady state. The linearization process excludes from the analysis possible noise-filtering mechanisms that show very sharp functions such as AND or OR gates [16] as well as oscillations or transitions between multiple steady states (such as the study on positive feedback loops and noise in [17]). Note also that the steady state *n*_0_ = *n*_1_ = *n*_2_ = 0 is not formally part of our analysis, because it renders the relative fluctuation *η* infinite.

Following linearization, the combined effect of all interaction parameters (i.e., Hill coefficients and saturation levels) is captured by the *elasticity* [3,13]:

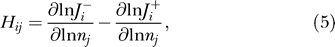
where 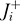 is the rate of generating *n_i_* (e.g., via transcription) and 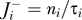 is its degradation rate. With these definitions, the susceptibility of the output was (Text S1, Section I)

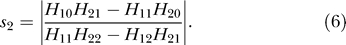
The absolute value facilitates a comparison between systems that increase or decrease their output when the input goes up.

Noise amplification was found by solving the matrix equation [3,13]

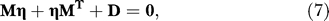
where the matrix **η** is composed of the normalized noise terms, the matrix **M** is related to the elasticities and time scales *τ_i_*, and the matrix **D** contains a single term corresponding to noise input from *n*_0_. In the construction of **D**, we assume that the sole noise source is fluctuations in *n*_0_, and that these fluctuations die out exponentially with a time scale *τ*_0_ = 1 (autocorrelation time of one unit). The exact terms of Equation 7 are defined in Section II of Text S1 and in [3,13].

A particular choice of elasticity values for all arrows of the network defines a single point in the 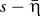 plane, and the general interplay between sensitivity and noise buffering was derived by considering a large number of different elasticity values (Figure 2A, Methods).

### Positive Feedback Is an Essential Component When Buffering Noise without a Reduction in Sensitivity

As expected, noise amplification in linear (unbranched) cascades is precisely proportional to the susceptibility (Figure 2B). This case of no feedback provides the reference for comparison for other network architectures. Consistent with the analysis above, in the case of a negative feedback, all the points appear *above* the reference line (Figure 2C) implying an increase in noise for a given level of susceptibility. Noise amplification at constant susceptibilities is observed also for the coherent (Figure 2D) and incoherent (Figure 2E) feed-forward loops (FFLs) [16,18], probably reflecting the addition of nonsynchronous noise components mediated through the intermediate node. In contrast, for positive feedback the points in the 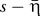 plot appear *below* the reference line (Figure 2F). Thus, for a given level of sensitivity, positive feedback buffers propagated noise.

To further characterize the properties of all three-node circuits, we calculated for each network the fraction of parameter sets that produce a stable steady state (Text S1, Section III), following the paradigm of [19]. We then calculated the fraction of stable parameter sets (Methods, Section IV of Text S1) that display high susceptibility and low noise (Figure 2G). Notably, networks that were stable throughout the entire parameter range provided poor noise buffering for a given susceptibility. None of these circuits contained positive feedback loops. In sharp contrast, the circuits that enhanced noise buffering were amenable to instability and were all composed of positive feedback loops. Taken together, among the networks tested, positive feedback appears to be required for buffering propagated noise while maintaining sensitivity.

### Positive Feedback Buffers Noise by Increasing Time Averaging

To better understand the reason underlying the ability of positive feedback to buffer propagated noise for a given susceptibility, we used the analytical description of a two-component system with an input *n*_0_ and an output *n*_1_, as was derived by Paulsson in [3,13] using the FDT approach [15]. In this framework (and while neglecting intrinsic noise), noise amplification was shown to be given by [3,13]:

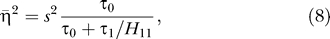
where *s* = −*H*_10_/*H*_11_ denotes the susceptibility; *τ*_0_, *τ*_1_ denote the degradation time scales of *n*_0_ and *n*_1_, respectively; and *H*_10_, *H*_11_ are the elasticities (as defined in Equation 5  above). In the absence of feedback, *H*_11_ = 1. Negative feedback of *n*_1_ on itself implies *H*_11_ > 1, whereas positive feedback implies 0 < *H*_11_ < 1 (if *H*_11_ falls below zero, instability arises). As was shown by Paulsson [3], negative feedback impairs noise buffering at a given susceptibility by effectively accelerating the dynamics and reducing averaging of fluctuations over time. Similarly, positive feedback enhances noise buffering for constant susceptibility by slowing down the dynamics and allowing for better time-averaging of fluctuating components.

Notably, Equation 8 suggests that positive feedback is not the only way to reduce *H*_11_ [3,13]. Moving beyond the first-order degradation assumed in our study, *H*_11_ can also be decreased if the degradation is independent of *n*_1_ (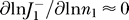 in Equation 5). This, in fact, is likely to be the case for nondividing microorganisms. Hence, time averaging would also be improved if the degradation is close to zero-order and the synthesis is not influenced by *n*_1_.

### A Noise-Buffering Circuit in the Control of Nutrient Homeostasis

While positive feedback appears to be important for buffering propagated noise (when sensitivity is controlled for), such a mechanism needs to comply with several requirements. First, the feedback loop itself should produce low internal noise because intrinsic noise is not buffered. Second, the effective elasticity *H*_11_ (Equation 8) should be of intermediate magnitude: when it is too high (*H*_11_ → 1) the effect of the positive feedback is negligible, but when it is too low (*H*_11_ → 0) the system is on the verge of instability, and the steady state will no longer resist small fluctuations. Finally, to avoid decrease in susceptibility due to saturation effects, *H*_11_ must be maintained constant over a large range of parameters.

**Figure 3:**
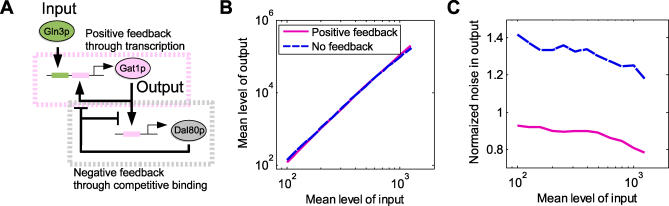
A Mechanism To Robustly Amplify a Signal and Average Noise (A) In the yeast nitrogen catabolite repression system, the transcription factor Gat1p is activated in response to Gln3p. It can then activate its own transcription, as well as DAL80, which binds to the same sequences as Gat1p and represses transcription. (B) The mean output (Gat1p) levels and its (C) noise content are shown for different input (nuclear Gln3p) levels. Simulation results are shown for the nitrogen system (pink line) and for a system with no feedback but with similar sensitivity (dashed blue line). The input noise level is *η*_0_ = 1, and its autocorrelation time is *τ*_0_ = 2 units. The other time constants are *τ*_1_ = *τ*_2_ =10 units. Dal80p binds its own promoter with a Hill coefficient of 2, whereas all other Hill coefficients equal 1.

A class of mechanisms that complies with the above requirements is based on a combination of positive and negative feedbacks. Fast-acting negative feedback functions to ensure stability, while positive feedback provides the required noise buffering. A specific example for such a network is involved in nitrogen homeostasis in yeast [20–22] (Figure 3A). Here, a transcription factor (Gat1p), which is activated by nuclear Gln3p, feeds back to enhance its own transcription, and in addition induces a transcriptional repressor (DAL80) that competes with Gat1p for the same DNA binding sites. This competition effectively weakens the positive feedback and ensures stability. Denoting the input signal to the system by *n*_0_, the output Gat1p by *n*_1_ and the repressor Dal80p by *n*_2_, the system can be modeled by the following two differential equations:

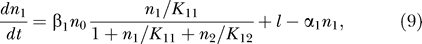


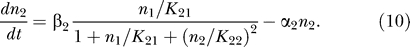



Here *α_i_* and *β_i_* denote the degradation and transcription rate constants, respectively, and *l* is a low rate of basal transcription required to prevent the shutdown of the system, *n*_1_ = *n*_2_ = 0. We will neglect this factor in subsequent analysis. The *K_ij_* coefficients in the protein production terms are dissociation constants, with *n*_2_/*K_i_*_2_ describing the competitive inhibition of Dal80p. The Hill coefficient of *n*_2_ binding to its own promoter is 2 because Dal80p binds as a dimer [21,22]. The Hill coefficient of *n*_2_ binding to the *n*_1_ promoter is set to 1 to enhance noise buffering and susceptibility (although a value of 2 would still increase noise averaging).

For the system described by Equations 9 and 10 to operate as a sensitive noise buffer, it must work in a regime where all interactions are unsaturated. Hence, all the binding constants of the repressor, *K_i_*_2_, must be small, and all binding constants of the activator, *K_i_*_1_, must be large. In this regime, Equation 9 and 10 reduce to

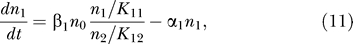
and

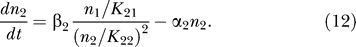
Finally, if *n*_2_ responds more rapidly than *n*_1_ and *n*_0_ (*H*_22_ *α*_2_ ≫ *H*_11_ *α*_1_, 1/*τ*_0_), then it can be assumed to be at quasi-steady state, and Equations 11  and 12 are combined to

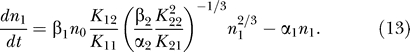
The power law dependence of the transcription rate on *n*_1_ results in an almost-constant elasticity 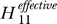 = 1/3 (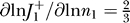 + in Equation 5). Hence, this network can buffer noise and maintain susceptibility for a large range of concentrations at which it remains unsaturated. A more rigorous analysis of the system is presented in Section VI of Text S1.

Detailed simulations confirm that this system can indeed buffer propagated noise, as compared to a loop-free system with the same levels of susceptibility (Figure 3B and 3C). Furthermore, the noise buffering capacity and the susceptibility of this system are maintained over a large range of input levels (Text S1, Section VI).

## Discussion

The ability to distinguish input signals from stochastic fluctuations is crucial for reliable information processing. Yet, being processed by the same computation device, signal and noise are inherently coupled. It thus comes as no surprise that increasing the ability to buffer propagated noise comes typically at the expense of reducing the sensitivity toward the input signal. We study this interplay in the context of a special class of systems where the signal is retained for long time periods, whereas the noise fluctuates rapidly. Such systems are ubiquitous in the adjustment of cells to aspects of their extracellular environment.

Previous studies reported that negative feedback buffers gene expression noise [10–12]. Nonetheless, when considering propagated noise that originates upstream of the feedback loop, this noise filtering merely reflects the reduction in the ability of the system to respond to changes in its input. Moreover, when parameters are chosen to preserve system sensitivity, negative feedback in fact amplifies, rather than reduces, propagated noise. By the same token, positive feedback, which appears to both increase the sensitivity of the system to changes in its input and to amplify intrinsic noise, reduces propagated noise when susceptibility (steady-state sensitivity) is controlled for.

Analytical analysis [3,13] revealed that noise propagation depends on two factors: the sensitivity to changes in input (susceptibility) on the one hand, and the averaging time [3,13] on the other hand. In the absence of feedback, this averaging time depends only on degradation rate. However, both negative and positive feedbacks impact this averaging time: negative feedback accelerates the dynamics [23] and consequently it reduces time averaging and does not buffer against noise. In contrast, positive feedback delays the kinetics leading to attenuation of propagated noise. If we view the feedback modules as low-pass frequency filters [12,24,25] and define a critical frequency [25] above which fluctuations are eliminated, then negative feedback increases this critical frequency, allowing more propagated noise to pass, whereas positive feedback decreases this frequency, thus reducing the amount of noise.

Whereas our study illustrates the effect of positive feedbacks, additional mechanisms could be used for reducing propagated noise by similarly increasing the averaging time. Such mechanisms include long linear cascades; cascades with an intermediate component that has a relatively large half-life [3,26]; or scenarios where both synthesis and degradation are essentially zero-order (from the definition of *H*_11_ in Equation 5). Finally, we note that systems that exhibit time delays together with bistability were not included in our screen but could also attenuate noise [17].

Positive feedbacks did not emerge as a recurrent network motif in several of the transcriptional networks analyzed [19]. One possibility is that designing the proper feedback that will maintain stability while providing noise buffering is evolutionarily difficult for the small size networks considered in these studies [19], due to the requirement it imposes on the extent of nonlinearities (Hill-coefficients) of the interactions. A simple realization of this concept, however, can easily be implemented by somewhat larger networks, as exemplified by the coupled positive–negative feedback we described. This and similar implementations function over a broad range of parameters and do not require strict tuning. Further analysis will be required to assess the abundance of this positive feedback–based noise-reduction scheme in different biological systems.

## Methods

### Simulations.

All simulations were based on the Gibson-Bruck [27] modification of Gillespie [28] algorithm. Input noise was implemented via transcription from a low copy mRNA with a short half life. No other mRNAs were explicitly considered. Simulation parameters are detailed in Section VII of Text S1. Simulations were carried out using Dizzy [29].

### Parameter screen.

The interaction parameters of each arrow in each network are captured by the interaction elasticities *H_ij_*. The susceptibility (Equation 2) for each network is calculated from the elasticities using Equation 6 (for a general derivation of the susceptibility, see Text S1, Section I). The noise amplification is connected to the elasticities through the solution of Equation 7. Definitions for the terms in Equation 7 appear in Section II of Text S1. Equation 7 was solved symbolically for all three node networks using Maple (MapleSoft, Waterloo Maple). Solutions for specific network architectures are shown in Table S1.

The values for the elasticities were randomly assigned to each network. To control for similar distribution of positive and negative interactions, we defined the synthesis elasticity *S_ij_* by *H_ii_* = 1 − *S_ii_* and *H_ij_* = −*S_ij_*. When *i* enhances the synthesis of *j*, then *S_ij_* > 0, and vice versa. Positive feedback of *i* on itself implies *S_ii_* > 0 and vice-versa. The synthesis elasticity values were sampled from a uniform distribution between zero and four and assigned to the arrows in each network. Different sampling ranges did not have a significant effect on the conclusions (Text S1, Section IV). We sampled 20,000 random sets of parameters for each circuit. The time constants were held fixed at a value of one, but different values did not change the results (Text S1, Section IV).

### Analysis of stability.

Stability criteria were established via the sign of the eigenvalues of the interaction matrix (Text S1, Section III).

## Supporting Information

Table S1 Properties of Several Networks (54 KB DOC)

Text S1 Mathematical and Numerical Details (280 KB PDF)

## Abbreviations

FFL: feed-forward loop
FDT: Fluctuation Dissipation Theorem

## References

[pcbi-0040008-b001] Bialek W, Setayeshgar S (2005). Physical limits to biochemical signaling. Proc Natl Acad Sci U S A.

[pcbi-0040008-b002] Maheshri N, O'Shea EK (2007). Living with noisy genes: how cells function reliably with inherent variability in gene expression. Annu Rev Biophys Biomol Struct.

[pcbi-0040008-b003] Paulsson J (2004). Summing up the noise in gene networks. Nature.

[pcbi-0040008-b004] Rao CV, Wolf DM, Arkin AP (2002). Control, exploitation and tolerance of intracellular noise. Nature.

[pcbi-0040008-b005] Raser JM, O'Shea EK (2005). Noise in gene expression: origins, consequences, and control. Science.

[pcbi-0040008-b006] Hooshangi S, Weiss R (2006). The effect of negative feedback on noise propagation in transcriptional gene networks. Chaos.

[pcbi-0040008-b007] Hooshangi S, Thiberge S, Weiss R (2005). Ultrasensitivity and noise propagation in a synthetic transcriptional cascade. Proc Natl Acad Sci U S A.

[pcbi-0040008-b008] Pedraza JM, van Oudenaarden A (2005). Noise propagation in gene networks. Science.

[pcbi-0040008-b009] Shibata T, Fujimoto K (2005). Noisy signal amplification in ultrasensitive signal transduction. Proc Natl Acad Sci U S A.

[pcbi-0040008-b010] Becskei A, Serrano L (2000). Engineering stability in gene networks by autoregulation. Nature.

[pcbi-0040008-b011] Dublanche Y, Michalodimitrakis K, Kümmerer N, Foglierini M, Serrano L (2006). Noise in transcription negative feedback loops: simulation and experimental analysis. Mol Syst Biol.

[pcbi-0040008-b012] Simpson ML, Cox CD, Sayler GS (2003). Frequency domain analysis of noise in autoregulated gene circuits. Proc Natl Acad Sci U S A.

[pcbi-0040008-b013] Paulsson J (2005). Models of stochastic gene expression. Phys Life Rev.

[pcbi-0040008-b014] Swain PS, Elowitz MB, Siggia ED (2002). Intrinsic and extrinsic contributions to stochasticity in gene expression. Proc Natl Acad Sci U S A.

[pcbi-0040008-b015] Keizer J (1987). Statistical thermodynamics of nonequilibrium processes.

[pcbi-0040008-b016] Mangan S, Zaslaver A, Alon U (2003). The coherent feedforward loop serves as a sign-sensitive delay element in transcription networks. J Mol Biol.

[pcbi-0040008-b017] Kim D, Kwon Y, Cho K (2006). Coupled positive and negative feedback circuits form an essential building block of cellular signaling pathways. BioEssays.

[pcbi-0040008-b018] Mangan S, Alon U (2003). Structure and function of the feed-forward loop network motif. Proc Natl Acad Sci U S A.

[pcbi-0040008-b019] Prill RJ, Iglesias PA, Levchenko A (2005). Dynamic properties of network motifs contribute to biological network organization. PLoS Biol.

[pcbi-0040008-b020] Boczko EM, Cooper TG, Gedeon T, Mischaikow K, Murdock DG (2005). Structure theorems and the dynamics of nitrogen catabolite repression in yeast. Proc Natl Acad Sci U S A.

[pcbi-0040008-b021] Cooper TG (2002). Transmitting the signal of excess nitrogen in Saccharomyces cerevisiae from the TOR proteins to the GATA factors: connecting the dots. FEMS Microbiol Rev.

[pcbi-0040008-b022] Magasanik B, Kaiser CA (2002). Nitrogen regulation in Saccharomyces cerevisiae. Gene.

[pcbi-0040008-b023] Rosenfeld N, Elowitz MB, Alon U (2002). Negative autoregulation speeds the response times of transcription networks. J Mol Biol.

[pcbi-0040008-b024] Austin DW, Allen MS, McCollum JM, Dar RD, Wilgus JR (2006). Gene network shaping of inherent noise spectra. Nature.

[pcbi-0040008-b025] Tan C, Reza F, You L (2007). Noise-limited frequency signal transmission in gene circuits. Biophys J.

[pcbi-0040008-b026] Doncic A, Ben-Jacob E, Barkai N (2006). Noise resistance in the spindle assembly checkpoint. Mol Syst Biol.

[pcbi-0040008-b027] Gibson MA, Bruck J (2000). Efficient exact stochastic simulation of chemical systems with many species and many channels. J Phys Chem A.

[pcbi-0040008-b028] Gillespie DT (1976). A general method for numerically simulating the stochastic time evolution of coupled chemical species. J Comp Phys.

[pcbi-0040008-b029] Ramsey S, Orrell D, Bolouri H (2005). Dizzy: stochastic simulation of large-scale genetic regulatory networks. J Bioinform Comput Biol.

