# Supplementary material for: Noise Propagation and Signaling Sensitivity in Biological Networks: A Role for Positive Feedback
Source: PLoS Comput Biol. 2008 Jan 4;4(1):e8. doi: 10.1371/journal.pcbi.0040008 (PMC2174979; doi:10.1371/journal.pcbi.0040008)
Supplement: Supplementary file 1 [file pcbi.0040008.sd001.pdf]

Noise propagation and signaling sensitivity in biological networks: A role for positive feedback loops

Supplementary Text

Contents

I. General Expression for Susceptibility 1

II. Expression for Noise Amplification 1

III. Criteria for Stability 2

IV. Design of Parameter Screen 2

V. Derivation of Noise and Susceptibility for a Two Component System 4
- A. Solution neglecting intrinsic noise 4
- B. Inclusion of Intrinsic Noise 5

VI. Biological Mechanisms that Filter Noise 6
- A. Mechanism 1 – Self positive feedback and activation of a repressor 8
- B. Mechanism 2 – Indirect positive feedback through a self-repressing component 9

VII. Simulation Parameters 9

References 12

I. GENERAL EXPRESSION FOR SUSCEPTIBILITY

We consider a network that is composed of N nodes that responds to an upstream input signal n_0. The system of equations that describe the kinetic response of the network is given by:

$$\frac{dn_i}{dt} = J_i^+(n_0, n_1, \ldots, n_N) - J_i^-(n_0, n_1, \ldots, n_N), \quad (1)$$

where J_i^+ and J_i^- are the total fluxes of production and elimination of n_i. At a stable steady state the equations given in (1) equal zero, hence

$$\left\langle J_i^+ \right\rangle = \left\langle J_i^- \right\rangle = \langle J_i \rangle \,. \quad (2)$$

The triangular brackets denote the steady state average.

The susceptibility of component j to changes in the input n_0 is

$$s_j = \frac{\langle n_0 \rangle}{\langle n_j \rangle} \frac{d \langle n_j \rangle}{d \langle n_0 \rangle} = \frac{d \ln \langle n_j \rangle}{d \ln \langle n_0 \rangle}. \quad (3)$$

Note that in the definition of susceptibility the derivatives are full derivatives, describing the changes in $\langle n_j \rangle$ after all the components have adjusted to the new steady state. Using the chain rule we differentiate Equation (1) at the steady state with respect to n_0, and then multiply by $\langle n_0 \rangle / \langle J_i \rangle$:

$$\begin{aligned} &\frac{\langle n_0 \rangle}{\langle J_i \rangle} \left(\frac{\partial \left\langle J_i^+ \right\rangle}{\partial \langle n_0 \rangle} - \frac{\partial \left\langle J_i^- \right\rangle}{\partial \langle n_0 \rangle} \right) + \\ &\frac{\langle n_1 \rangle}{\langle J_i \rangle} \left(\frac{\partial \left\langle J_i^+ \right\rangle}{\partial \langle n_1 \rangle} - \frac{\partial \left\langle J_i^- \right\rangle}{\partial \langle n_1 \rangle} \right) \frac{\langle n_0 \rangle}{\langle n_1 \rangle} \frac{d \langle n_1 \rangle}{d \langle n_0 \rangle} + \ \cdots \\ &+ \frac{\langle n_N \rangle}{\langle J_i \rangle} \left(\frac{\partial \left\langle J_i^+ \right\rangle}{\partial \langle n_N \rangle} - \frac{\partial \left\langle J_i^- \right\rangle}{\partial \langle n_N \rangle} \right) \frac{\langle n_0 \rangle}{\langle n_N \rangle} \frac{d \langle n_N \rangle}{d \langle n_0 \rangle} \ = \ 0. \quad (4) \end{aligned}$$

Finally using Equation (2), we obtain

$$H_{i0} + H_{i1} s_1 + \ldots + H_{iN} s_N = 0, \quad (5)$$

where the s_j terms are the susceptibility of each individual component in the network (Equation (3)) and the H_{ij} terms are the reaction flux elasticities, as defined by Paulsson [8, 9]

$$\begin{aligned} H_{ij} &= -\frac{\langle n_j \rangle}{\langle J_i \rangle} \left(\frac{\partial \left\langle J_i^+ \right\rangle}{\partial \langle n_j \rangle} - \frac{\partial \left\langle J_i^- \right\rangle}{\partial \langle n_j \rangle} \right) \\ &= \frac{\partial \ln \left\langle J_i^- \right\rangle / \left\langle J_i^+ \right\rangle}{\partial \ln \langle n_j \rangle}. \end{aligned} \quad (6)$$

In matrix form:

$$\mathbf{H}\vec{s} = -\vec{k}, \quad (7)$$

where the terms of $\mathbf{H}$ are H_{ij} and the terms of $\vec{k}$ are H_{i0}.

II. EXPRESSION FOR NOISE AMPLIFICATION

We derived an analytical expression for noise amplification in a three component system using Paulsson's FDT-based approach [9]. Using this method the normalized (standard deviation over mean) noise components are given by the matrix equation

$$\mathbf{M}\eta + \eta \mathbf{M}^T + \mathbf{D} = 0, \quad (8)$$

where the matrix η contains the normalized noise terms

$$\eta = \begin{pmatrix} \eta_0^2 & \eta_{01} & \eta_{02} \\ \eta_{10} & \eta_1^2 & \eta_{12} \\ \eta_{20} & \eta_{21} & \eta_2^2 \end{pmatrix}. \tag{9}$$

The matrix $\mathbf{M}$ describes the effect that each component exerts on its neighbours

$$\mathbf{M} = \begin{pmatrix} -1/\tau_0 & 0 & 0 \\ -H_{10}/\tau_1 & -H_{11}/\tau_1 & -H_{12}/\tau_1 \\ -H_{20}/\tau_2 & -H_{21}/\tau_2 & -H_{22}/\tau_2 \end{pmatrix}. \tag{10}$$

The H_{ij} terms are the elasticities (Equation (6)) and the τ_i parameters are the degradation time scales for each component. Note that the first row of $\mathbf{M}$ contains only one non-zero term, because components 1 and 2 do not affect the input.

The matrix $\mathbf{D}$ is composed of the noise sources. We assume that most of the noise originates from the input n_0, and hence $\mathbf{D}$ has only one non-zero term:

$$\mathbf{D} = \begin{pmatrix} 2\eta_0^2/\tau_0 & 0 & 0 \\ 0 & 0 & 0 \\ 0 & 0 & 0 \end{pmatrix}. \tag{11}$$

Equation (8) was solved symbolically for η_2^2 using Maple (Maplesoft). The solution was used to substitute different values of H_{ij} and characterize the susceptibility-noise amplification relation for each network architecture.

III. CRITERIA FOR STABILITY

In this section we derive criteria for stability of a system with one input node, n_0, and two other components n_1, n_2. The differential equations for this system are

$$\frac{dn_1}{dt} = J_1^+(n_1, n_2|n_0) - J_1^-(n_1, n_2|n_0), \tag{12a}$$

and

$$\frac{dn_2}{dt} = J_2^+(n_1, n_2|n_0) - J_2^-(n_1, n_2|n_0). \tag{12b}$$

We distinguish n_0 from n_1 and n_2 because it can affect n_1 and n_2 but is not affected by them, and is therefore treated as a parameter of the system. The Jacobian for this system of equations is given by

$$\mathbf{A} = \begin{pmatrix} \left[\frac{\partial J_1^+}{\partial n_1} - \frac{\partial J_1^-}{\partial n_1}\right] & \left[\frac{\partial J_1^+}{\partial n_2} - \frac{\partial J_1^-}{\partial n_2}\right] \\ \left[\frac{\partial J_2^+}{\partial n_1} - \frac{\partial J_2^-}{\partial n_1}\right] & \left[\frac{\partial J_2^+}{\partial n_2} - \frac{\partial J_2^-}{\partial n_2}\right] \end{pmatrix}, \tag{13}$$

and the eigenvalues of the Jacobian conform to

$$(A_{11} - \lambda)(A_{22} - \lambda) - A_{12}A_{21} = 0. \tag{14}$$

For the system to have a stable solution the eigenvalues must be negative. Therefore, the components of the Jacobian must satisfy

$$A_{11} + A_{22} < 0, \tag{15a}$$

and

$$A_{11}A_{22} - A_{12}A_{21} > 0. \tag{15b}$$

The Jacobian is related to the elasticities through $A_{ij} = -H_{ij}J_i/n_j$ (Equation (6)). With this, the criteria for stability becomes

$$H_{11}/\tau_1 + H_{22}/\tau_2 > 0, \tag{16a}$$

and

$$H_{11}H_{22} - H_{12}H_{21} > 0. \tag{16b}$$

with the τ_i terms defined as the time scales of the reactions $\tau_i = n_i/J_i^-$.

IV. DESIGN OF PARAMETER SCREEN

Each network is defined by the connections between its nodes and by the sign of the connections, *i.e.* activation or repression. We require a connection from n_0 to n_1 and from n_1 to n_2. Each of these connections can either be repressing or activating. There are four additional possible connections in the network ($n_0 \to n_2$, $n_1 \to n_2$, $n_1 \to n_1$, $n_2 \to n_2$) which can be repressing, activating or non-existing. Thus we investigated $2^2 \times 3^4 = 324$ specific circuits.

If node i is affected by node j then their interaction is captured by the equation

$$\frac{dn_i}{dt} = J_i^+(\ldots, n_j, \ldots) - J_i^-(\ldots, n_j, \ldots). \tag{17}$$

To calculate the susceptibility and noise properties of a specific network some information must be provided on the magnitude of the fluxes J_i^+ and J_i^- near the steady state, specifically their elasticities (see Sections I and II, and Equation (6)).

For the purposes of the screen we assume that the components undergo first order degradation, $\partial \ln \langle J_i^- \rangle / \partial \ln \langle n_j \rangle = 1$. With this assumption the effect of one component on the other is captured by the *synthesis elasticity*, defined as the relative change in the steady state transcription rate of n_i due to a small percent change in the level of n_j

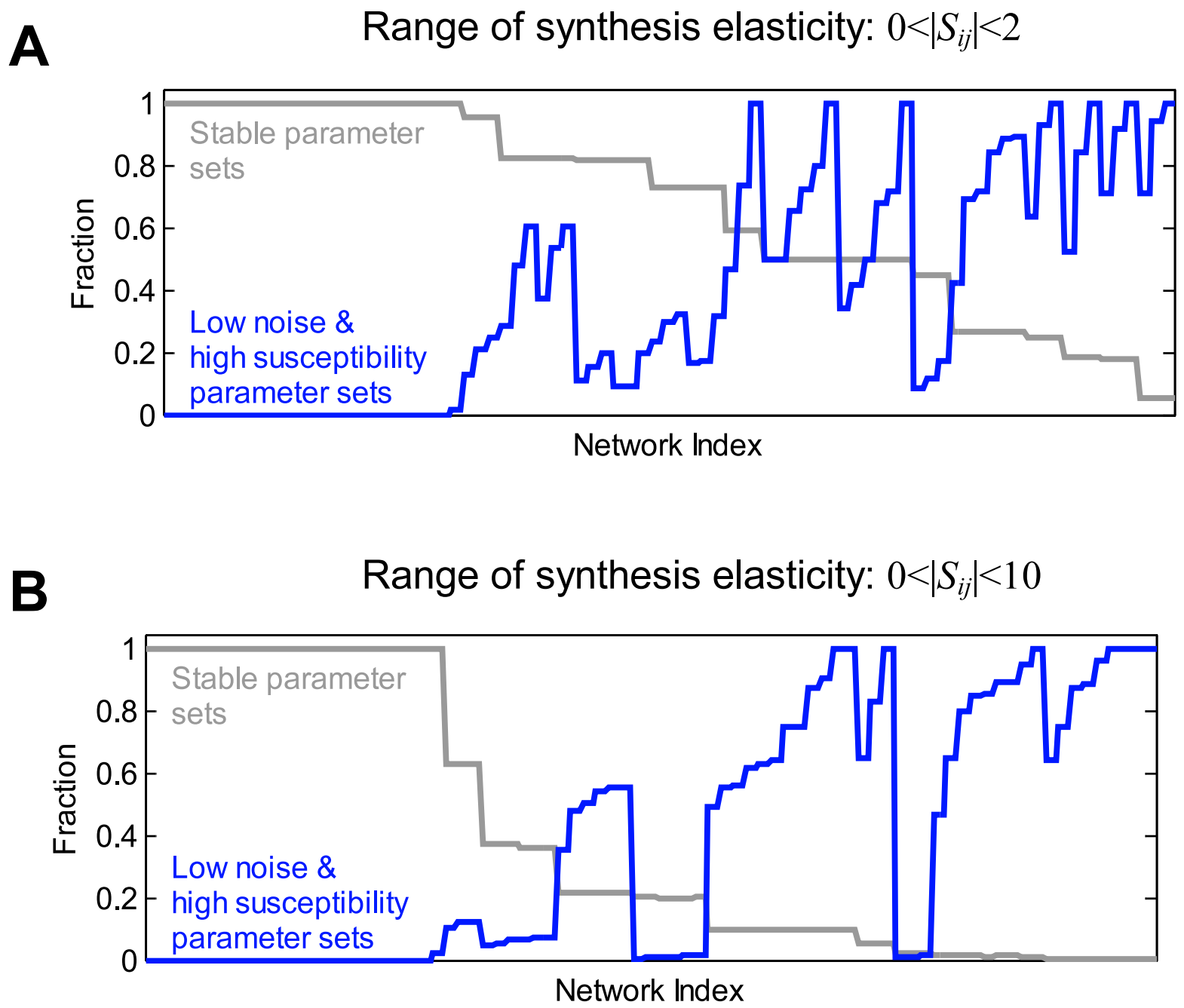

FIG. 1: All three-node circuits we investigated were sorted into groups according to decreasing fraction of stable parameter sets out of all parameters sets (gray line). Within each group the networks were sorted according to the fraction of highly sensitive low-noise parameter sets out of all stable sets (blue line). The results are shown for a sampling range **(A)** $0 < |S_{ij}| < 2$ and **(B)** $0 < |S_{ij}| < 10$.

$$S_{ij} = \frac{\langle n_j \rangle}{\langle J_i^+ \rangle} \frac{\partial \langle J_i^+ \rangle}{\partial \langle n_j \rangle} = \frac{\partial \ln \langle J_i^+ \rangle}{\partial \ln \langle n_j \rangle}. \tag{18}$$

The synthesis elasticities are related to the overall reaction elasticities through $H_{ii} = 1 - S_{ii}$ and $H_{ij} = -S_{ij}$. We chose to work with the synthesis elasticities because a positive S_{ij} would always indicate that n_i enhances n_j (even when $i = j$) and vice versa (assuming first order degradation). In this framework the sensitivity of the interaction between i and j is determined through the absolute value $|S_{ij}|$ and the nature of the interaction (activation or repression) is described by the sign of the interaction arrow of the network. This makes the sampling procedure of the parameter space much simpler. Furthermore, the synthesis elasticities have an intuitive physical meaning – they are proportional to the Hill coefficient of transcription factor binding and they decrease as the saturation increases.

To assess the susceptibility-noise amplification behavior of each network we considered many (20,000) positive values for S_{ij}. The sampling distribution of S_{ij} could, in principle, have an effect on the results. To generate Fig. 2 of the main text we sampled S_{ij} from a uniform distribution between zero and four (chosen arbitrarily). Here we test the effect of the sampling range on the results. Supplementary Fig. 1 presents the fraction of stable parameter sets and the fraction of high-susceptibility low-noise parameters sets, for each network in the screen. The figure is presented for two sampling ranges, $0 < |S_{ij}| < 2$ and $0 < |S_{ij}| < 10$. The sampling range affects the stability as well as the fraction of high-susceptibility low-noise parameter sets. Nevertheless, regardless of the sampling range, all circuits with perfect stability do not display high susceptibility and low noise. Furthermore, the networks that exhibit the best noise properties (highest fraction of parameter sets with low noise amplification at a given susceptibility) contain only positive feedback loops (a combination of a coherent feed-forward element in addition to three positive feedback loops is also possible in the sampling range $0 < |S_{ij}| < 10$, possibly because the noise buffering capacity of the three positive feedback loops overwhelms the slight increase in noise due to the feed-forward). Hence, changing the sampling range of the parameter screen does not impact the qualitative conclusions from the screen.

In the design of the screen we also set the time scales of all components of the networks equal to one. A choice of different values for the time scales alters the slope of the linear relation between noise amplification and susceptibility for linear networks (Supplementary Fig. 2a). Nonetheless, the fraction of parameter sets that lie above or below this linear line is virtually unaffected by the choice of the time constants (Supplementary Fig. 2b).

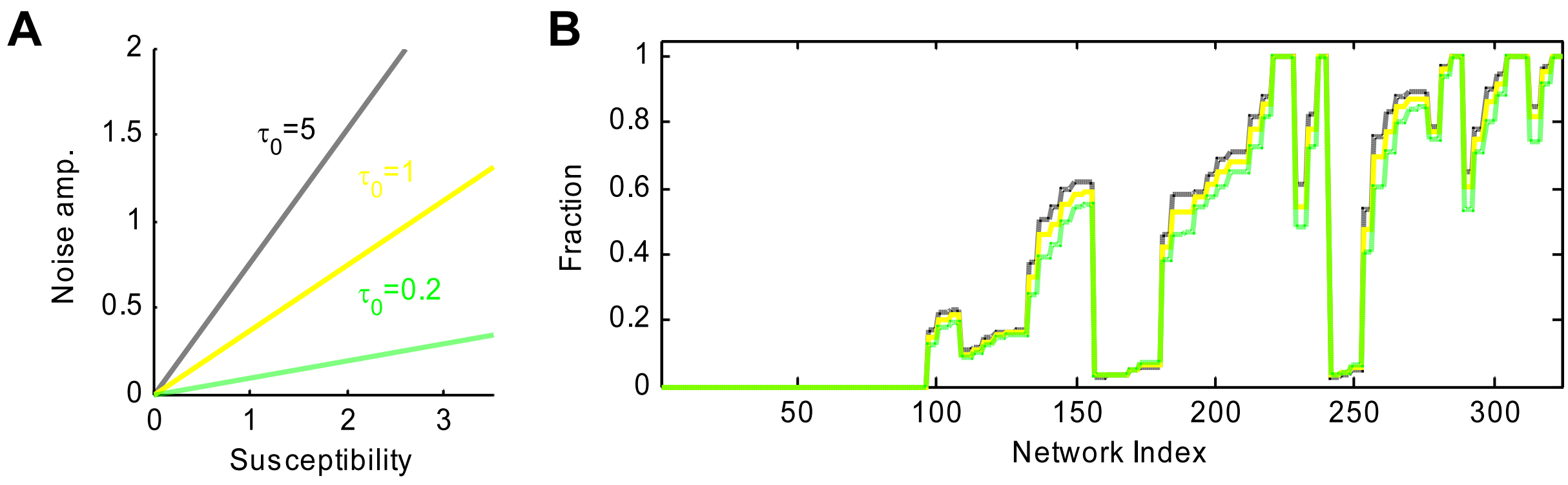

FIG. 2: **(A)** The relation between susceptibility and noise amplification for linear networks, when the input noise autocorrelation time τ_0 is varied. **(B)** Fraction of parameter sets that show low noise amplification at constant susceptibility (compared to the linear networks), for each network in the screen. Different colors represent different τ_0 as in (A).

V. DERIVATION OF NOISE AND SUSCEPTIBILITY FOR A TWO COMPONENT SYSTEM

The noise properties of a two component circuit were derived by Paulsson [8, 9] using Fluctuation Dissipation Theorem [3]. For the sake of completeness we bring a similar derivation based on Frequency Domain Analysis [7, 12].

A. Solution neglecting intrinsic noise

We consider the simplest gene network: a two component system with an input n_0 and output n_1. We derive the susceptibility of n_1 to changes in n_0, and the magnitude of noise that propagates from n_0 to n_1. The system is described by a single differential equation

$$\frac{dn_1}{dt} = J^+(n_0, n_1) - J^-(n_0, n_1), \tag{19}$$

where J^+ is flux of generation of n_1, and J^- is the flux of elimination. Typically, the elimination term will follow first order kinetics $J^-(n_0, n_1) = const \times n_1$, but we will consider the more general case. The average steady state levels of n_0 and n_1 are given by $\langle n_0 \rangle$ and $\langle n_1 \rangle$. To find the change in n_1 steady state levels due to a small change in n_0, we repeat the steps in Section I, and differentiate Equation (19) with respect to n_0 at the steady state:

$$\left(\frac{\partial \langle J^+\rangle}{\partial \langle n_0\rangle} - \frac{\partial \langle J^-\rangle}{\partial \langle n_0\rangle}\right) + \left(\frac{\partial \langle J^+\rangle}{\partial \langle n_1\rangle} - \frac{\partial \langle J^-\rangle}{\partial \langle n_1\rangle}\right)\frac{d\langle n_1\rangle}{d\langle n_0\rangle} = 0. \tag{20}$$

Recall that at steady state $\langle J^+\rangle = \langle J^-\rangle = \langle J\rangle$. To obtain an expression for the susceptibility $s_1 = \frac{\langle n_0\rangle}{\langle n_1\rangle}\frac{d\langle n_1\rangle}{d\langle n_0\rangle}$ we multiply Equation (20) by $\langle n_0\rangle / \langle J\rangle$ and rearrange to get

$$s_1 = -\frac{H_{10}}{H_{11}}. \tag{21}$$

The elasticities H_{10} and H_{11} are defined by

$$H_{10} = \frac{\partial \ln \langle J^-\rangle / \langle J^+\rangle}{\partial \ln \langle n_0\rangle}, \tag{22a}$$

and

$$H_{11} = \frac{\partial \ln \langle J^-\rangle / \langle J^+\rangle}{\partial \ln \langle n_1\rangle}. \tag{22b}$$

When $H_{10} > 0$ then n_0 *downregulates* n_1 and vice versa. First-order degradation of n_1 with no feedback implies $H_{11} = 1$. Positive feedback of n_1 on itself is characterized by $H_{11} < 1$, and negative feedback results in $H_{11} > 1$.

Now we turn to derive the level of noise that is propagated from n_0 to n_1. The approach we use for this purpose is frequency domain analysis (see [7, 12] and the supplementary of [1, 10]). We linearize Equation (19), and rewrite it in terms of fluctuations from the steady state $\Delta n_j(t) = n_j(t) - \langle n_j \rangle$:

$$\frac{d\Delta n_1}{dt} = \left(\frac{\partial \langle J^+\rangle}{\partial \langle n_0\rangle} - \frac{\partial \langle J^-\rangle}{\partial \langle n_0\rangle}\right)\Delta n_0 + \left(\frac{\partial \langle J^+\rangle}{\partial \langle n_1\rangle} - \frac{\partial \langle J^-\rangle}{\partial \langle n_1\rangle}\right)\Delta n_1.$$

Normalizing the equation through division by $\langle J\rangle$ we arrive at

$$\frac{\langle n_1\rangle}{\langle J\rangle}\frac{dx_1}{dt} = \frac{\langle n_0\rangle}{\langle J\rangle}\left(\frac{\partial \langle J^+\rangle}{\partial \langle n_0\rangle} - \frac{\partial \langle J^-\rangle}{\partial \langle n_0\rangle}\right)x_0 + \frac{\langle n_1\rangle}{\langle J\rangle}\left(\frac{\partial \langle J^+\rangle}{\partial \langle n_1\rangle} - \frac{\partial \langle J^-\rangle}{\partial \langle n_1\rangle}\right)x_1.$$

The x_j variables are the normalized deviation from the steady state,

$$x_j(t) = \frac{n_j(t) - \langle n_j \rangle}{\langle n_j \rangle} \qquad j = \{0, 1\}. \tag{23}$$

In terms of elasticities we get

$$-\tau_1 \frac{dx_1}{dt} = H_{10} x_0 + H_{11} x_1, \tag{24}$$

with τ_1 describing the time constant of n_1 turnover, and it is defined as

$$\tau_1(\langle n_0 \rangle, \langle n_1 \rangle) = \frac{\langle n_1 \rangle}{\langle J \rangle}.$$

We apply the Fourier transform to Equation (24) and move from the time domain to the frequency domain:

$$\hat{x}_j(\omega) = \int_{-\infty}^{\infty} x_j(t) e^{-i\omega t} \mathrm{d}t \qquad j = \{0, 1\}, \tag{25}$$

resulting in the relation

$$\hat{x}_1(\omega) = -\frac{H_{10}}{H_{11}} \frac{1}{i\,\tau_{mod}\,\omega + 1}\, \hat{x}_0(\omega), \tag{26}$$

where τ_{mod} is the time constant modified by the feedback $\tau_{mod} = \tau_1/H_{11}$. The function $H_{10}/H_{11}\,(i\,\tau_{mod}\,\omega + 1)^{-1}$ in Equation (26) is sometimes referred to as the transfer function [2] or the impulse response function [7], because it relates the frequency response of x_1 to that of the input. To describe the noise in x_0 and x_1 we utilize the concept of the autocorrelation function $\langle x_j(t_0) x_j(t_0 + t') \rangle$. We assume the fluctuations x_0 can be represented by an exponentially decreasing autocorrelation function, with magnitude η_0^2,

$$\langle x_0(t_0)\, x_0(t_0 + t') \rangle = \eta_0^2 e^{-t'/\tau_0}. \tag{27}$$

The Fourier transform of the autocorrelation function of x_0 (which is termed the *power spectrum* of x_0) is

$$\langle \hat{x}_0(\omega)\, \hat{x}_0^*(\omega) \rangle = 2\eta_0^2 \frac{\tau_0}{\tau_0^2 \omega^2 + 1}. \tag{28}$$

The asterisk denotes the complex conjugate. The power spectrum of x_1 can be derived by multiplying $\hat{x}_1(\omega)$ from Equation (26) by its conjugate and taking the ensemble average [2, 7]. Then, using Equation (28)

$$\begin{aligned} \langle \hat{x}_1(\omega)\, \hat{x}_1^*(\omega) \rangle &= \frac{H_{10}^2}{H_{11}^2} \frac{1}{(\tau_{mod}\,\omega)^2 + 1}\, \langle \hat{x}_0(\omega)\, \hat{x}_0^*(\omega) \rangle \\ &= 2\eta_0^2 \frac{H_{10}^2}{H_{11}^2} \frac{1}{(\tau_{mod}\,\omega)^2 + 1} \frac{\tau_0}{(\tau_0 \omega)^2 + 1} \end{aligned} \tag{29}$$

To find the magnitude of the noise η_1 we return to the time domain and substitute $t' = 0$ in the autocorrelation function:

$$\begin{aligned} \eta_1^2 &= \frac{1}{2\pi} \int_{-\infty}^{\infty} \langle \hat{x}_1(\omega)\, \hat{x}_1^*(\omega) \rangle\, e^{i\omega t'} \mathrm{d}\omega \bigg|_{t'=0} \\ &= \frac{1}{2\pi} \int_{-\infty}^{\infty} \langle \hat{x}_1(\omega)\, \hat{x}_1^*(\omega) \rangle\, \mathrm{d}\omega. \end{aligned} \tag{30}$$

Solving the integral in (30), substituting the result for the susceptibility (Equation (21)) and recalling that $\tau_{mod} = \tau_1/H_{11}$ we arrive at

$$\eta_1^2/\eta_0^2 = s_1^2 \frac{\tau_0}{\tau_0 + \tau_1/H_{11}}. \tag{31}$$

In the case of a loop-free cascade and first order degrdation $H_{11} = 1$. If there is a negative feedback on n_1 then $H_{11} > 1$ and the noise is amplified beyond that of the linear cascade. However, when the feedback is positive and $0 < H_{11} < 1$ (which may still allow for the existence of a stable steady state) then the noise amplification can be much lower than that of the linear cascade, for a given susceptibility.

B. Inclusion of Intrinsic Noise

The analysis above of a system composed of a single input and a single output did not take into account intrinsic noise that arises from translational bursts [6, 13] or other sources [8, 11]. In this section we incorporate, following [6, 13], noise that arises from the production of low-copy short-lived mRNA, denoted by m:

$$\frac{dm}{dt} = \beta_m(n_0, n_1) - m/\tau_m + f_m(t). \tag{32a}$$

The protein n_1 is translated from the mRNA:

$$\frac{dn_1}{dt} = \beta\, m - n_1/\tau_1 + f(t). \tag{32b}$$

The terms β_m, β, τ_m^{-1} and τ_1^{-1} are the production and degradation rates of the mRNA and the protein, respectively. β_m depends on n_0 and n_1 because these proteins may in general modulate the transcription rate of n_1. The functions $f_m(t)$ and $f(t)$ are white noise terms that have a mean zero and a very short autocorrelation time:

$$\langle f_m(t) \rangle = 0, \tag{33a}$$

$$\langle f_m(t_0) f_m(t_0 + t') \rangle = q_m \delta(t' - t_0), \tag{33b}$$

and similarly for $f(t)$

$$\langle f(t_0) f(t_0 + t') \rangle = q\delta(t' - t_0). \tag{33c}$$

The prefactors q_m and q define the magnitude of the autocorrelation for the fluctuations in the mRNA and in n_1, respectively. They are determined by the molecular interactions [3]. Typically the noise originates from a random birth-death process, hence its magnitude is the sum of the mean reaction fluxes [3, 6, 14]:

$$q_m = \beta_m \left(\langle n_0 \rangle , \langle n_1 \rangle \right) + \langle m \rangle / \tau_m = 2 \langle m \rangle / \tau_m, \quad (34a)$$

and

$$q = \beta \langle m \rangle + \langle n_1 \rangle / \tau_1 = 2 \langle n_1 \rangle / \tau_1. \quad (34b)$$

As before, we linearize Equations (32a) and (32b) near the mean steady state and divide by the steady state fluxes $\beta_m = \langle m \rangle / \tau_m$ and $\beta \langle m \rangle = \langle n_1 \rangle / \tau_1$ to obtain

$$\tau_m \frac{dx_m}{dt} = -H_{m1} x_1 - H_{m0} x_0 - x_m + \frac{\tau_m f_m(t)}{\langle m \rangle}, \quad (35a)$$

and

$$\tau_1 \frac{dx_1}{dt} = x_m - x_1 + \frac{\tau_1 f(t)}{\langle n_1 \rangle}. \quad (35b)$$

The elasticities H_{mj} for $j = \{0, 1\}$ are given by

$$H_{mj} = -\frac{\langle n_j \rangle}{\beta_m} \frac{\partial \beta_m}{\partial \langle n_j \rangle}, \quad (36)$$

and the normalized deviations from the steady state are defined as $x_j = (n_j - \langle n_j \rangle) / \langle n_j \rangle$ for $j = \{0, 1, m\}$. Performing the Fourier transform on Equations (35a) and (35b) we arrive at

$$(i\tau_m \omega + 1)\, \hat{x}_m = -H_{m1} \hat{x}_1 - H_{m0} \hat{x}_0 + \frac{\tau_m \hat{f}_m(\omega)}{\langle m \rangle} \quad (37a)$$

and

$$(i\tau_1 \omega + 1)\, \hat{x}_1 = \hat{x}_m + \frac{\tau_1 \hat{f}(\omega)}{\langle n_1 \rangle}. \quad (37b)$$

If we are interested in correlation times that are much longer than the mRNA degradation time, $t' \gg \tau_m$, then $\tau_m \omega \ll 1$. In this case the solution for $\hat{x}_1(\omega)$, which can be found from (37a) and (37b), reduces to

$$\begin{aligned} \hat{x}_1(\omega) &= -\frac{H_{m0}}{i\tau_1\omega + H_{11}} \hat{x}_0 + \frac{\tau_m \hat{f}_m(\omega)}{(i\tau_1\omega + H_{11}) \langle m \rangle} \\ &\quad + \frac{\tau_1 \hat{f}(\omega)}{(i\tau_1\omega + H_{11}) \langle n_1 \rangle}, \end{aligned} \quad (38)$$

with the definition $H_{11} = 1 + H_{m1}$. Equation (38) describes how fluctuations are transferred from each noise source to x_1. We multiply $\hat{x}_1(\omega)$ in equation (38) by its conjugate and then perform an ensemble average [2, 7]. Because all noise sources are uncorrelated their cross-correlation terms equal zero. The power spectrum of $\hat{f}_m(\omega)$ and $\hat{f}(\omega)$ can be derived from the Fourier transform of Equations (33c), (34a) and (34b). The resulting expression for the power spectrum of x_1 is

$$\begin{aligned} \langle \hat{x}_1(\omega) \hat{x}_1^*(\omega) \rangle &= \frac{H_{m0}^2}{\tau_1^2\omega^2 + H_{11}^2} \langle \hat{x}_0(\omega) \hat{x}_0^*(\omega) \rangle + \\ &\quad \frac{2\tau_m}{(\tau_1^2\omega^2 + H_{11}^2) \langle m \rangle} + \\ &\quad \frac{2\tau_1}{(\tau_1^2\omega^2 + H_{11}^2) \langle n_1 \rangle}. \end{aligned} \quad (39)$$

As explained in Equations (27) and (28) in the previous section, we assume that the autocorrelation of x_0 decreases exponentially with a time constant τ_0. Hence

$$\langle \hat{x}_0(\omega)\, \hat{x}_0^*(\omega) \rangle = 2\eta_0^2 \frac{\tau_0}{\tau_0^2\omega^2 + 1}. \quad (40)$$

The normalized noise in n_1 can be found from the power spectrum of x_1 as explained in Equation (30) of the previous section:

$$\begin{aligned} \eta_1^2 &= \frac{1}{2\pi} \int_{-\infty}^{\infty} \langle \hat{x}_1(\omega)\, \hat{x}_1^*(\omega) \rangle \, \mathrm{d}\omega \\ &= \frac{H_{m0}^2}{H_{11}^2} \frac{\tau_0}{\tau_0 + \tau_1/H_{11}} \eta_0^2 + \frac{\tau_m}{\tau_1 H_{11} \langle m \rangle} + \frac{1}{H_{11} \langle n_1 \rangle}. \end{aligned}$$

We recall that the susceptibility is $s_1 = -H_{m0}/H_{11}$, and define the burst coefficient as the average number of proteins that are produced from a single mRNA molecule, $b = \beta\tau_m = \tau_m \langle n_1 \rangle / \tau_1 \langle m \rangle$. Finally we arrive at

$$\eta_1^2 = \frac{1 + b}{H_{11} \langle n_1 \rangle} + s_1^2 \frac{\tau_0}{\tau_0 + \tau_1/H_{11}} \eta_0^2. \quad (41)$$

The contribution of the intrinsic noise is captured by the term $(1 + b) / (H_{11} \langle n_1 \rangle)$.

VI. BIOLOGICAL MECHANISMS THAT FILTER NOISE

Positive feedback that increases susceptibility and time averaging occurs at intermediate elasticities, *i.e.* $0 < H_{11} < 1$ in Equation (31). To preserve this behavior over a large range of input levels a constant value of H_{11} must be maintened. One possible biological mechanism that can retain $0 < H_{11} < 1$ is positive feedback that involves negative cooperativity (where one subunit of the protein inhibits the binding of a second subunit, leading to Hill coefficients lower than one). Although negative cooperativity was found in signal transduction pathways

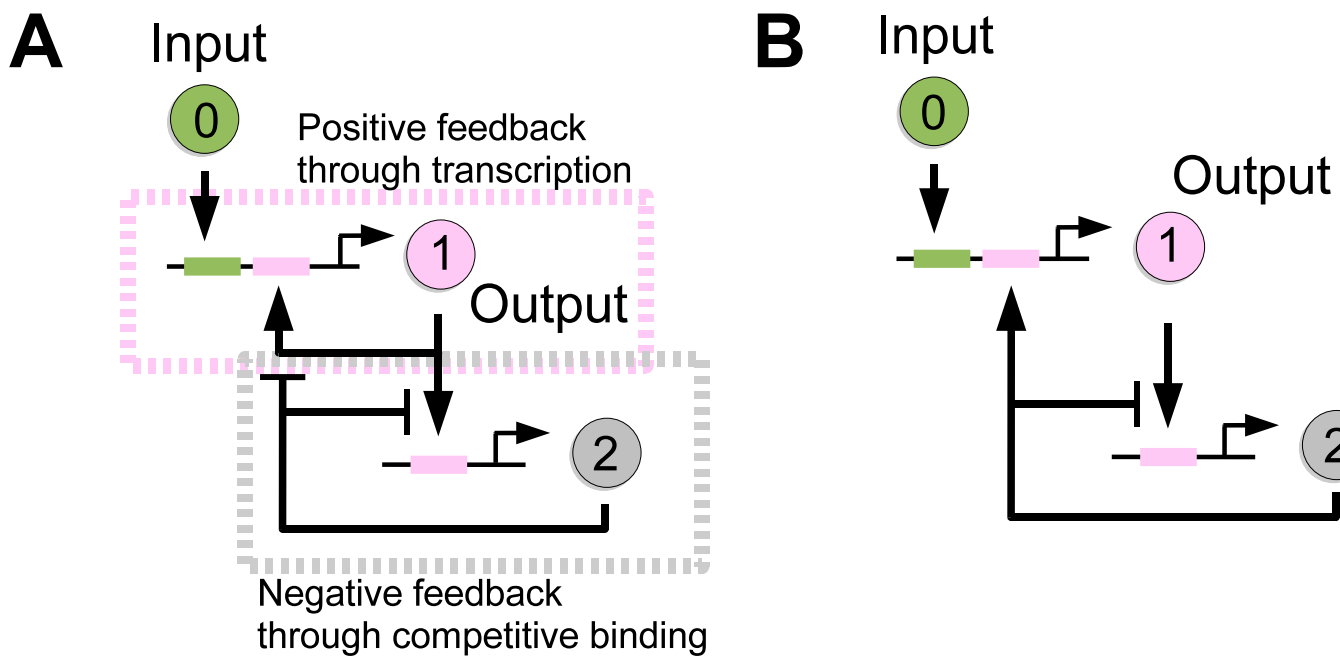

FIG. 3: Mechanisms to generate positive feedback that filters noise. **(A)** A transcription factor 1 responds to an input 0 and activates its own transcription. It also activates the transcription of a repressor 2 that competes with the transcription factor binding. **(B)** An input signal 0 activates the transcription of 1, which activates 2, that can also activate 1, closing a positive feedback loop. Protein 2 also represses its own transcription by competing with 1.

[4, 5], it was not described in transcriptional networks, to the best of our knowledge.

In this section we suggest a class of transcriptional networks that can provide high sensitivity and low noise amplification over a large range of input levels. These networks include positive feedback combined with competitive inhibition-based negative feedback. The competitive inhibition effectively decreases the binding affinity and generates effective negative cooperativity. The proposed mechanisms are shown in Supplementary Fig. 3. Before we analyze each case separately, we derive a general formulae for the susceptibility and noise amplification of the systems in Supplementary Fig. 3.

As derived in Section I the susceptibility vector is given by

$$\mathbf{H}\vec{s} = -\vec{k}, \tag{42}$$

where the components of $\mathbf{H}$ are the elasticities H_{ij} and the terms of $\vec{k}$ are H_{i0}. For the class of three component systems at hand

$$\mathbf{H} = \begin{pmatrix} H_{11} & H_{12} \\ H_{21} & H_{22} \end{pmatrix} \tag{43a}$$

and

$$\vec{k} = \begin{pmatrix} H_{10} \\ 0 \end{pmatrix}. \tag{43b}$$

The susceptibility of component n_1 to changes in the input n_0 will be

$$s_1 = -\frac{H_{10}}{H_{11} - H_{12}H_{21}/H_{22}}. \tag{44}$$

To find the noise amplification we use Paulsson's FDT-based approach ([9] and Section II). Using this method the matrix of normalized noise components, η, is given by

$$\mathbf{M}\eta + \eta\mathbf{M}^T + \mathbf{D} = 0, \tag{45}$$

where

$$\mathbf{M} = \begin{pmatrix} -\alpha_0 & 0 & 0 \\ -\alpha_1 H_{10} & -\alpha_1 H_{11} & -\alpha_1 H_{12} \\ 0 & -\alpha_2 H_{21} & -\alpha_2 H_{22} \end{pmatrix} \tag{46a}$$

and

$$\mathbf{D} = \begin{pmatrix} 2\alpha_0\eta_0^2 & 0 & 0 \\ 0 & 0 & 0 \\ 0 & 0 & 0 \end{pmatrix}, \tag{46b}$$

where the α_i terms are the degradation rates of each component. After some algebra the noise amplification is given by

$$\begin{aligned} \frac{\eta_1^2}{\eta_0^2} &= s_1^2 \times \frac{\alpha_1\left(H_{11} - H_{12}H_{21}/H_{22}\right)}{\alpha_0 + \alpha_1\left(H_{11} - H_{12}H_{21}/H_{22}\right)} \times \\ & \frac{\alpha_0 + \alpha_1\left(H_{11} - H_{12}H_{21}/H_{22}\right)}{\alpha_0 + \alpha_1\left(H_{11} - \frac{\alpha_2 H_{22}}{\alpha_0 + \alpha_2 H_{22}}\frac{H_{12}H_{21}}{H_{22}}\right)} \times \\ & \left(1 - \frac{\alpha_0}{\alpha_0 + \alpha_2 H_{22}}\frac{\alpha_1}{\alpha_1 H_{11} + \alpha_2 H_{22}}\frac{H_{12}H_{21}}{H_{22}}\right) \end{aligned} \tag{47}$$

The first two terms in the equation are the susceptibility and "ideal" time averaging (compare these terms to Equation (31)). The elasticity H_{11} is modified by a factor $H_{12}H_{21}/H_{22}$. This captures the effects of inhibition through n_2. The last two terms of the equation are correction terms that arise because the initiation of n_2 is not immediate. These terms approach one when the response time of the intermediate component n_2 is very short. From this analysis we immediately note that in order to have good noise properties two requirements must

be fulfilled: First, the Hill coefficients should be adjusted such that the elasticities will conform to

$$0 < H_{11} - H_{12}H_{21}/H_{22} < 1, \tag{48}$$

and second, that component n_2 will respond quickly

$$\alpha_2 H_{22} \gg \alpha_1 H_{11}. \tag{49}$$

We now discuss each of the specific examples in Supplementary Fig. 3 separately.

A. Mechanism 1 – Self positive feedback and activation of a repressor

In the first mechanism of Supplementary Fig. 3a, an input signal, labeled 0, activates the output gene, labeled 1. Gene 1 encodes for a transcription factor that can directly enhance its own transcription. Transcription factor 1 also activates a repressor 2, that can bind to the same promoters as 1, thereby competitively inhibiting it. The differential equations that describe this system are:

$$\frac{dn_1}{dt} = \beta_1 n_0 \frac{n_1/K_{11}}{1 + n_1/K_{11} + n_2/K_{12}} + l - \alpha_1 n_1, \tag{50a}$$

$$\frac{dn_2}{dt} = \beta_2 \frac{n_1/K_{21}}{1 + n_1/K_{21} + n_2/K_{22}} - \alpha_2 n_2. \tag{50b}$$

where the β_i terms denote the transcription rate coefficients and the K_{ij} terms are the Michaelis-Menten binding constants. l is some low-level basal transcription which is needed to avoid the solution $n_1 = n_2 = 0$. In the next derivations we neglect l. From these equations the elasticities are given by

$$H_{10} = -1, \tag{51a}$$

$$H_{11} = 1 - \frac{1 + n_2/K_{12}}{1 + n_1/K_{11} + n_2/K_{12}}, \tag{51b}$$

$$H_{12} = \frac{n_2/K_{12}}{1 + n_1/K_{11} + n_2/K_{12}}, \tag{51c}$$

$$H_{21} = -\frac{1 + n_2/K_{22}}{1 + n_1/K_{21} + n_2/K_{22}}, \tag{51d}$$

and

$$H_{22} = 1 + \frac{n_2/K_{22}}{1 + n_1/K_{21} + n_2/K_{22}}. \tag{51e}$$

Note that all Hill coefficients in this specific example equal one. Different Hill coefficients can be used just as long as the requirement in Equation (48) is fulfilled. In Fig. 3 of the main text, for example, the repressor binds its own promoter with a Hill coefficient of 2, whereas all other Hill coefficients equal 1.

Another requirement from the positive feedback, such that it can buffer noise effectively, is that it will not saturate over a large range of input signals, otherwise the susceptibility will vanish. This necessitates strong repression

$$\frac{n_2}{K_{12}} \gg \frac{n_1}{K_{11}}, \tag{52a}$$

$$\frac{n_2}{K_{22}} \gg \frac{n_1}{K_{21}}, \tag{52b}$$

$$\frac{n_2}{K_{12}} \gg 1, \tag{52c}$$

and

$$\frac{n_2}{K_{22}} \gg 1. \tag{52d}$$

Using (52a), (52b), (52c) and (52d) in (50a) and (50b) at the steady-state, we find that

$$n_1 \approx \frac{\left(\frac{\beta_1}{\alpha_1}\frac{K_{12}}{K_{11}}\right)^2}{\frac{\beta_2}{\alpha_2}\frac{K_{22}}{K_{21}}} n_0^2, \tag{53a}$$

and

$$n_2 \approx \left(\frac{\beta_1}{\alpha_1}\frac{K_{12}}{K_{11}}\right) n_0. \tag{53b}$$

The upper bound on n_0, above which the system saturates, is given by substituting (53a) and (53b) back into the conditions in (52a) and (52b):

$$\left(\frac{\beta_1/\alpha_1}{\beta_2/\alpha_2}\frac{K_{12}}{K_{11}}\right) n_0 \ll 1. \tag{54a}$$

and

$$\left(\frac{\beta_1/\alpha_1}{\beta_2/\alpha_2}\left(\frac{K_{12}}{K_{11}}\right)^2 \frac{K_{21}}{K_{22}}\right) n_0 \ll 1. \tag{54b}$$

The lower bound follows from Equations (52c) and (52d), but the system will probably be limited by intrinsic noise when the number of molecules is too low.

The expressions in (54a) and (54b) simply mean that in order to maintain strong repression over a large range of input signals, the transcription level of the repressor n_2 and its affinity to the promoters should be large compared to that of transcription factor n_1. This is the third condition, beyond Equations (48) and (49), that would make this system a good noise filter.

B. Mechanism 2 – Indirect positive feedback through a self-repressing component

Mechanism 2 is presented in Supplementary Fig. 3. The input signal n_0 activates the transcription of the output protein n_1. The protein n_1 can activate the transcription of n_2. The latter has two functions (it can also be two different proteins), on the one hand it enhances the transcription of the output n_1, thus establishing the positive feedback loop. On the other hand n_2 represses its own transcription through competitive binding, thereby reducing the effect of the positive feedback. The kinetic equations for this system are

$$\frac{dn_1}{dt} = \beta_1 n_0 \frac{n_2/K_{12}}{1+n_2/K_{12}} - \alpha_1 n_1, \tag{55a}$$

$$\frac{dn_2}{dt} = \beta_2 \frac{n_1/K_{21}}{1+n_1/K_{21}+n_2/K_{22}} - \alpha_2 n_2. \tag{55b}$$

The corresponding elasticities are

$$H_{10} = -1, \tag{56a}$$

$$H_{11} = 1, \tag{56b}$$

$$H_{12} = -\frac{1}{1+n_2/K_{12}}, \tag{56c}$$

$$H_{21} = -\frac{1+n_2/K_{22}}{1+n_1/K_{21}+n_2/K_{22}}, \tag{56d}$$

and

$$H_{22} = 1 + \frac{n_2/K_{22}}{1+n_1/K_{21}+n_2/K_{22}}. \tag{56e}$$

The values of the elasticities can be manipulated by varying the Hill coefficients (which were taken to be 1 in this example), just as long as the relation in Equation (48) is kept.

Using similar arguments as in Section VI A we arrive at the scaling of n_1 and n_2 when the system is far from saturation:

$$n_1 \approx \left(\left(\frac{\beta_1/\alpha_1}{K_{12}} \right)^2 \frac{\beta_2/\alpha_2 K_{22}}{K_{21}} \right) n_0^2, \tag{57a}$$

and

$$n_2 \approx \left(\frac{\beta_1/\alpha_1}{K_{12}} \frac{\beta_2/\alpha_2 K_{22}}{K_{21}} \right) n_0. \tag{57b}$$

Consequently, the upper bounds on n_0 to maintain an unsaturated response are

$$\left(\frac{\beta_1/\alpha_1}{K_{12}} \frac{K_{22}}{K_{21}} \right) n_0 \ll 1, \tag{58a}$$

and

$$\left(\frac{\beta_1}{\alpha_1} \frac{\beta_2}{\alpha_2} \frac{K_{22}}{K_{12}^2 K_{21}} \right) n_0 \ll 1. \tag{58b}$$

Simulations have shown that this mechanism can indeed buffer noise when compared to a linear cascade with the same susceptibility (not shown).

VII. SIMULATION PARAMETERS

The reactions and parameters that were simulated in the figures of the main text are given below.

Description	Reaction	Rate
Generate mRNA	$\phi \to m_0$	β_{m0}
Degrade mRNA	$m_0 \to \phi$	$\alpha_{m0} m_0$
Generate n_0	$\phi \to n_0$	$\beta_0 m_0$
Degrade n_0	$n_0 \to \phi$	$\alpha_0 n_0$
Generate n_1 include feedback	$\phi \to n_1$	$\beta_1 \frac{n_0}{K+n_0} \left(K_1^h + n_1^h\right)^{-1}$
Generate n_1 no feedback	$\phi \to n_1$	$\beta_1 \frac{n_0}{K+n_0}$
Degrade n_1	$n_1 \to \phi$	$\alpha_1 n_1$

TABLE I: Reactions simulated in Fig. 1 of the main text

Parameter	Value	
	No feedback	Feedback
h	—	4
α_{m0}	5	5
α_0	0.1	0.1
α_1	0.1	0.1
β_{m0}	1.5	1.5
β_0	67	67
β_1	120	1.92×10^{11}
K	1000	1000
K_1	10	10
n_0	200	200

TABLE II: Parameters for the simulation in Fig. 1c of the main text

Parameter	Value		
h	0	1	2
α_{m0}	5	5	5
α_0	0.5	0.5	0.5
α_1	0.1	0.1	0.1
β_{m0}	5	5	5
β_0	Adjusted according to n_0		
β_1	225	1.52×10^4	1.77×10^6
K	1000	1000	1000
K_1	10	10	10
n_0	Mean from 125 to 8000		

TABLE III: Parameters for the simulation in Fig. 1d of the main text

Description	Reaction	Rate
Generate mRNA	$\phi \to m_0$	β_{m0}
Degrade mRNA	$m_0 \to \phi$	$\alpha_{m0} m_0$
Generate n_0	$\phi \to n_0$	$\beta_0 m_0$
Degrade n_0	$n_0 \to \phi$	$\alpha_0 n_0$
Generate n_1	$\phi \to n_1$	$\beta_1 n_0 \frac{n_1/K_{11}}{1+n_1/K_{11}+n_2/K_{12}} + l$
Degrade n_1	$n_1 \to \phi$	$\alpha_1 n_1$
Generate n_2	$\phi \to n_2$	$\beta_2 \frac{n_1/K_{21}}{1+n_1/K_{21}+(n_2/K_{22})^2}$
Degrade n_2	$n_2 \to \phi$	$\alpha_2 n_2$
Generate n_1 - no feedback	$\phi \to n_1$	$c\, n_0^r$

TABLE IV: Reactions simulated in Fig. 3 of the main text

Parameter	Value
α_{m0}	5
α_0	0.5
α_1	0.1
α_2	0.1
β_{m0}	0.45
β_0	Adjusted according to n_0
β_1	200
β_2	8×10^5
K_{11}	10^4
K_{12}	10
K_{21}	10^4
K_{22}	10
l	0.1
n_0	Mean value from 100 to 1260
r, c	Adjusted to the steady state and susceptibility of the system with the feedback

TABLE V: Parameters for the simulation in Fig. 3 of the main text

[1] D. W. Austin, M. S. Allen, J. M. McCollum, R. D. Dar, J. R. Wilgus, G. S. Sayler, N. F. Samatova, C. D. Cox, and M. L. Simpson. Gene network shaping of inherent noise spectra. *Nature*, 439:608–11, Feb 2006.

[2] J. C. Doyle, B. A. Francis, and A. R. Tannenbaum. *Feedback Control Theory.* Macmillian, `http://www.control.utoronto.ca/people/profs/francis/dft.html`, 1992.

[3] J. Keizer. *Statistical thermodynamics of nonequilibrium processes.* Springer-Verlag, 1987.

[4] A. Levitzki and Koshland D. E. Jr. Negative cooperativity in regulatory enzymes. *Proc. Natl. Acad. Sci. USA*, 62(4):1121–8, 1969.

[5] P. De Meyts, E. Van Obberghen, J. Roth, A. Wollmer, and D. Brandenburg. Mapping of the residues responsible for the negative cooperativity of the receptor-binding region of insulin. *Nature*, 273:504–9, 1978.

[6] E. M. Ozbudak, M. Thattai, I. Kurtser, A. D. Grossman, and A. van Oudenaarden. Regulation of noise in the expression of a single gene. *Nat. Genet.*, 31:69–73, May 2002.

[7] A. Papoulis. *Probability, Random Variables and Stochastic Processes.* McGraw-Hill, 1991.

[8] J. Paulsson. Summing up the noise in gene networks. *Nature*, 427:415–8, Jan 2004.

[9] J. Paulsson. Models of stochastic gene expression. *Phys. Life Rev.*, 2:157–175, 2005.

[10] J. M. Pedraza and A. van Oudenaarden. Noise propagation in gene networks. *Science*, 307:1965–9, Mar 2005.

[11] J. M. Raser and E. K. O'Shea. Control of stochasticity in eukaryotic gene expression. *Science*, 304:1811–4, Jun 2004.

[12] M. L. Simpson, C. D. Cox, and G. S. Sayler. Frequency domain analysis of noise in autoregulated gene circuits. *Proc. Natl. Acad. Sci. USA*, 100:4551–6, Apr 2003.

[13] M. Thattai and A. van Oudenaarden. Intrinsic noise in gene regulatory networks. *Proc. Natl. Acad. Sci. USA*, 98:8614–9, Jul 2001.

[14] M. Thattai and A. van Oudenaarden. Attenuation of noise in ultrasensitive signaling cascades. *Biophys. J.*, 82:2943–50, Jun 2002.
