# Supplementary material for: Noise Propagation and Signaling Sensitivity in Biological Networks: A Role for Positive Feedback
Source: PLoS Comput Biol. 2008 Jan 4;4(1):e8. doi: 10.1371/journal.pcbi.0040008 (PMC2174979; doi:10.1371/journal.pcbi.0040008)
Supplement: Supplementary file 2 [file pcbi.0040008.st001.doc]

# Supplementary Table S1 - Properties of several networks

| **Motif name** | **Drawing** | **Responsiveness** | **Noise gain** | **Remarks** |
| --- | --- | --- | --- | --- |
| Straightforward cascade |  | $s_{2}=H_{10}H_{21}$ | $\frac{\eta_{2}^{2}}{\eta_{0}^{2}}=s_{2}^{2}\frac{\alpha_{1}\alpha_{2}\left(\alpha_{0}+\alpha_{1}+\alpha_{2}\right)}{\left(\alpha_{0}+\alpha_{1}\right)\left(\alpha_{0}+\alpha_{2}\right)\left(\alpha_{1}+\alpha_{2}\right)}$ |  |
| Fast autoregulation |  | $s_{2}=\frac{H_{10}H_{21}}{H_{11}H_{22}}$ | $\frac{\eta_{2}^{2}}{\eta_{0}^{2}}=s_{2}^{2}\frac{\alpha_{1}H_{11}\alpha_{2}H_{22}\left(\alpha_{0}+\alpha_{1}H_{11}+\alpha_{2}H_{22}\right)}{\left(\alpha_{0}+\alpha_{1}H_{11}\right)\left(\alpha_{0}+\alpha_{2}H_{22}\right)\left(\alpha_{1}H_{11}+\alpha_{2}H_{22}\right)}$ | For negative autoregulation $H_{ii}>1$, for positive autoregulation $H_{ii}<1$. |
| Feed-forward loop |  | $s_{2}=H_{10}H_{21}-H_{20}$ | $\begin{matrix} \frac{\eta_{2}^{2}}{\eta_{0}^{2}}=s_{2}^{2}\frac{\alpha_{1}\alpha_{2}\left(\alpha_{0}+\alpha_{1}+\alpha_{2}\right)}{\left(\alpha_{0}+\alpha_{1}\right)\left(\alpha_{0}+\alpha_{2}\right)\left(\alpha_{1}+\alpha_{2}\right)}+ \\ \begin{matrix} & \end{matrix}H_{20}^{2}\frac{\alpha_{0}\alpha_{2}^{2}}{\left(\alpha_{0}+\alpha_{1}\right)\left(\alpha_{0}+\alpha_{2}\right)\left(\alpha_{1}+\alpha_{2}\right)} \end{matrix}$ | When the feed-forward is coherent then the responsiveness increases in comparison to the straightforward cascade. When the feed-forward is incoherent the responsiveness decreases. In both cases there is a residual noise term which is independent of the responsiveness. |
| Two-node feedback |  | $s_{2}=\frac{H_{10}H_{21}}{1-H_{12}H_{21}}$ | $\begin{matrix} \frac{\eta_{2}^{2}}{\eta_{0}^{2}}=s_{2}^{2}\frac{\alpha_{1}\alpha_{2}\left(\alpha_{0}+\alpha_{1}+\alpha_{2}\right)}{\left(\alpha_{0}+\alpha_{1}\right)\left(\alpha_{0}+\alpha_{2}\right)\left(\alpha_{1}+\alpha_{2}\right)}\times\\ \begin{matrix} & & \end{matrix}\frac{1-H_{12}H_{21}}{1-\frac{\alpha_{1}}{\alpha_{0}+\alpha_{1}}\frac{\alpha_{2}}{\alpha_{0}+\alpha_{2}}H_{12}H_{21}} \end{matrix}$ | When the feedback is negative $H_{12}H_{21}<0$and the last term of the noise gain is larger than one. This term is lower than one when the feedback is positive and $0<H_{12}H_{21}<1$. |

The *α_i_* terms are the degradation rates of each component.

The Hij terms are elasticities, defined as

$H_{ij}=\frac{\partial\ln\left\langle J_{i}^{-}\right\rangle/\left\langle J_{i}^{+}\right\rangle}{\partial\ln\left\langle n_{j}\right\rangle}$.

When *H_ij_*>0 then *n_j_* **downregulates** *n_i_* and vice versa.
